# Elevated Risk of Acute Urine Retention in Patients with Symptomatic Benign Prostate Hyperplasia Following Coronavirus Disease 2019 Infection: A Retrospective Cohort Study from TriNetX

**DOI:** 10.3390/life16050729

**Published:** 2026-04-25

**Authors:** Jen-Chieh Lin, Cheng-Hua Lee, Jheng-Yan Wu, Wen-Hsin Tseng, Chien-Liang Liu, Steven K. Huang, Allen W. Chiu

**Affiliations:** 1 Division of Urology, Department of Surgery, Chi Mei Medical Center, Tainan 71004, Taiwan; 0440lsda@gmail.com (J.-C.L.);; 2 School of Medicine, College of Medicine, Chung Shan Medical University, Taichung 40201, Taiwan; 3 Department of Nutrition, Chi Mei Medical Center, Tainan 71004, Taiwan; andy10271@gmail.com; 4 Department of Public Health, College of Medicine, National Cheng Kung University, Tainan 70101, Taiwan; 5 Institute of Biomedical Science, National Sun Yat-Sen University, Kaohsiung 80424, Taiwan; 6 Division of Uro-Oncology, Department of Surgery, Chi Mei Medical Center, Tainan 71004, Taiwan; 7 Department of Urology, Shin Kong Wu Ho-Su Memorial Hospital, Taipei 11008, Taiwan

**Keywords:** BPH, lower urinary tract symptoms, COVID-19

## Abstract

Purpose: To investigate the association between COVID-19 infection and the 1-year risk of acute urinary retention (AUR) and related urological complications in patients with benign prostatic hyperplasia (BPH) and lower urinary tract symptoms (LUTs). Materials and Methods: Using the TriNetX global network, patients with BPH and LUTs between January 2020 and January 2024 were identified. Participants were classified into a COVID-19 cohort (N = 32,948) and a non-COVID control cohort (N = 434,123). Propensity score matching (1:1) balanced demographics, comorbidities, medications, and laboratory parameters. The primary outcome was AUR within one year. Secondary outcomes included Foley catheterization, urinary tract infection (UTI), gross hematuria, bladder stones, and prostate-related surgery. Results: After matching, 32,918 patients remained in each cohort. The COVID-19 group showed a significantly higher 1-year incidence of AUR compared with controls (2.18% vs. 0.32%; aHR 6.89, 95% CI 5.62–8.45; *p* < 0.0001). Increased risks were also observed for Foley catheterization (aHR 4.10), UTI (aHR 3.52), and prostate-related surgery (aHR 6.02). Kaplan–Meier analysis demonstrated persistent divergence in AUR-free survival. Conclusion: COVID-19 infection is independently associated with a markedly increased risk of AUR and urological complications in patients with BPH, highlighting the need for closer post-infection monitoring.

## 1. Introduction

Benign prostatic hyperplasia (BPH) is a highly prevalent condition among aging men, characterized by progressive enlargement of periurethral prostatic tissue resulting from an imbalance between cellular proliferation and apoptosis [1,2,3]. Its prevalence increases steadily with age, affecting approximately half of men over 50 years and more than 80% of those older than 70 years [2,4]. Lower urinary tract symptoms (LUTs), including urinary frequency, urgency, nocturia, and weak urinary stream, substantially impair quality of life and contribute to an increasing healthcare burden in men with BPH, especially aging populations [5,6].

Acute urinary retention (AUR) is the most common urological emergency worldwide and represents one of the most severe complications of BPH-related bladder outlet obstruction (BOO) [7,8]. It occurs predominantly in older men and is most frequently caused by prostatic enlargement leading to mechanical obstruction of urinary flow [7,8]. The risk of AUR increases with advancing age and is further influenced by factors such as increased prostate volume, elevated prostate-specific antigen levels, infection, medication effects, and systemic comorbidities [9,10]. Because AUR often necessitates emergent catheterization, hospitalization, and subsequent intervention, its prevention remains a key therapeutic goal in the management of patients with BPH and LUTs [7,11].

Since the World Health Organization (WHO) declared COVID-19 a public health emergency on January 30, 2020, a growing body of evidence has linked SARS-CoV-2 infection to various urinary complications, most notably acute urinary retention [12,13]. In a large retrospective cohort of 834 hospitalized COVID-19 patients, Avilez et al. reported that 12.6% of patients requiring urinary catheterization developed sustained urinary retention, with greater pulmonary involvement and disease severity associated with prolonged catheter dependence and failed catheter removal [14]. In patients with pre-existing BPH, Pavlović et al. demonstrated that COVID-19 infection was followed by significant worsening of LUTs, including increases in prostate-specific antigen levels, prostate volume, and post-void residual urine, with residual urine identified as a significant predictor of AUR development [15]. In addition, Saleh et al. described urinary retention as an unusual presenting manifestation of COVID-19, occurring in the context of severe hyponatremia and presumed neuroinvasive effects, highlighting that urinary retention may arise through heterogeneous mechanisms beyond simple mechanical obstruction [16].

Despite these observations, existing studies are limited by small sample sizes, heterogeneous patient populations, and predominantly retrospective or descriptive designs. Moreover, few large-scale studies have specifically evaluated the long-term risk of acute urinary retention among patients with pre-existing BPH following COVID-19 infection. Therefore, this study aimed to investigate the association between COVID-19 infection and the subsequent risk of AUR in patients with BPH using a large, multicenter real-world database. By applying propensity score matching to balance baseline characteristics and comorbidities, we evaluated the 1-year risk of AUR and other urological outcomes in patients with and without a history of COVID-19 infection.

## 2. Materials and Methods

### 2.1. Data Source

We conducted this retrospective study using TriNetX, which comprises approximately 190 million de-identified electronic health records from 170 healthcare organizations (HCOs). Available information included demographics, diagnoses based on ICD-10 (International Classification of Diseases, 10th edition), procedures based on the current procedural terminology codes, medications based on the RxNorm codes, laboratory tests, and health care utilization records. Our study initially included 74,145,690 patients with ≥2 HCO visits from 30 January 2020 to 30 January 2024. This study was approved by the Institutional Review Board (IRB) (Number 11302-E01, approved on 24 January 2024) of Chi Mei Medical Center, which also waived the informed consent. The study complied with the principles of the Declaration of Helsinki and adhered to the Strengthening the Reporting of Observational Studies in Epidemiology (STROBE) reporting guideline. Artificial intelligence tools were used to assist with grammar correction and language editing.

### 2.2. Study Design and Sample Size Calculation

**Figure 1:**
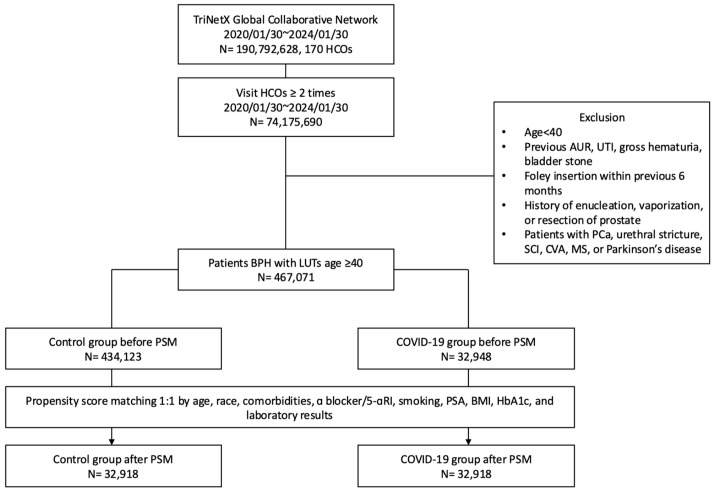
Flow chart. 5-αRI: 5-Alpha Reductase Inhibitor; AUR: Acute Urinary Retention; BMI: Body Mass Index; BPH: Benign Prostatic Hyperplasia; COVID-19: Coronavirus Disease 2019; CVA: Cerebrovascular Accident; HbA1c: Hemoglobin A1c; HCOs: Healthcare Organizations; LUTs: Lower Urinary Tract Symptoms; MS: Multiple Sclerosis; PCa: Prostate Cancer; PSA: Prostate-Specific Antigen; PSM: Propensity Score Matching; SCI: Spinal Cord Injury; UTI: Urinary Tract Infection; α blocker: Alpha-Adrenergic Blocker.

On 30 January 2020, the World Health Organization (WHO) declared the COVID-19 outbreak a public health emergency of international concern (PHEIC) [12]. As shown in Figure 1, between 30 January 2020 and 30 January 2024, a total of 74,175,690 patients with ≥2 HCOs visits were initially enrolled. Exclusion criteria consisted of age under 40, history of AUR), urinary tract infection (UTI), gross hematuria, bladder stones, prostate cancer, urethral stricture, spinal cord injury (SCI), cerebrovascular accident (CVA), multiple sclerosis (MS), Parkinson’s disease (PD), Foley catheter insertion within the previous 6 months, and prior prostate surgery (enucleation, vaporization, or resection). The sample size was not pre-determined by power analysis, given the retrospective nature of the study using a global federated database. Due to the lack of missing data imputation in the TriNetX platform, we restricted our analyses to patients with complete records for all study variables. A cohort of 467,071 patients aged ≥40 years with benign prostatic hyperplasia and lower urinary tract symptoms (ICD-10: N40.1) was included. They were divided into a COVID-19 group (N = 32,948) and a control group (N = 434,123) based on infection history, defined by a diagnosis of COVID-19 (ICD-10: U07.1) or a positive PCR test (TNX curated 9088). After 1:1 propensity score matching (PSM) for age, race, comorbidities, α blockers/5-αRIs, smoking, PSA, BMI, HbA1c, and laboratory results, 32,918 patients were finally identified for each group. Detailed definitions and coding of each group are available in Table S1 of the Supplementary Materials.

### 2.3. Covariates

To balance the cohorts, PSM was employed using a comprehensive set of baseline variables. Demographics encompassed age at index and race. Clinical markers included prostate-specific antigen (PSA), body mass index (BMI), hemoglobin A1c (HbA1c), systolic blood pressure, estimated glomerular filtration rate (eGFR), low-density lipoprotein (LDL), and total cholesterol. Comorbid conditions were adjusted for hypertensive diseases, diabetes mellitus (DM), overweight/obesity, tobacco use, and ischemic heart disease. Additionally, the use of ɑ-blocker and 5-ɑ reductase inhibitors were also accounted for. Detailed variable definitions and coding are available in Table 1 and Table S2 of the Supplementary Materials.

### 2.4. Outcomes and Follow-Up

The primary outcome was acute urine retention (ICD-10 code: R33) as the primary priority. Secondary outcomes included Foley insertion (CPT: 51702 or ICD-10: Z96.0), urinary tract infection (ICD-10: N39.0), gross hematuria (ICD-10: R31.0), bladder stones (ICD-10: N21.0), and prostate-related surgery (ICD-10-PCS: 0VT08ZZ, CPT: 52648, or CPT: 52649). To exclude immediate postoperative complications and minimize baseline interference, the follow-up window began one month after the index date and continued for up to one year. Additional details regarding specific outcome definitions and procedural coding are provided in Table S3 of the Supplementary Materials.

### 2.5. Statistical Analysis

Descriptive statistics were employed to summarize the data, with continuous variables expressed as means ± standard deviations and categorical variables reported as counts and percentages. PSM was performed with the aforementioned covariates. A standardized mean difference (SMD) of ≤0.1 was set as the threshold for achieving well balance. To evaluate the risks for each outcome, Cox proportional hazards regression models were utilized to calculate adjusted hazard ratios (aHRs) with 95% confidence intervals (CIs). The proportional hazards assumption was assessed and confirmed using Schoenfeld residuals. The Altman and Bland method was used to estimate P for interaction to determine whether the effect of COVID-19 on AUR risk differed significantly between subgroups, with a *p*-value < 0.05 considered statistically significant. To provide a more rigorous assessment of potential unmeasured confounding, we reported the E-value based on the lower limit of the 95% confidence interval. Furthermore, the 1-year AUR-free survival between 2 groups was analyzed via Kaplan–Meier curves and compared using log-rank tests.

### 2.6. Subgroup Analysis

Subgroup analyses were stratified by age (≥65 VS. <65), hypertension (HTN) (presence vs. absence), DM (presence vs. absence), tobacco use (smoker vs. non-smoker), alpha blocker (user vs. non-user), 5-alpha reductase inhibitor (user vs. non-user), BMI (≥27 vs. <27, kg/m^2^), and eGFR (≥45 vs. <45, mL/min/1.73 m^2^).

## 3. Results

### 3.1. Baseline Characteristics of Included Patients

Table 1 summarizes the baseline characteristics of the study cohorts. Prior to matching, the mean age in the COVID-19 group was slightly higher than in the control group (66.65 vs. 65.93 years old). In terms of racial distribution, the COVID-19 cohort featured a larger proportion of White patients compared to the control group (76.0% vs. 62.0%), while the control group had a notably higher percentage of patients with unknown race (20.9%).

As for baseline comorbidities, patients in the COVID-19 group exhibited a higher prevalence of hypertensive diseases (38.2% vs. 19.3%), diabetes mellitus (20.4% vs. 9.7%), ischemic heart diseases (13.1% vs. 5.8%), and overweight (8.7% vs. 3.0%). Similarly, a higher utilization percentage of alpha blockers (16.9% vs. 9.3%) and 5-alpha reductase inhibitors (4.3% vs. 2.4%) was also observed in the COVID-19 group. Laboratory findings at baseline showed that the COVID-19 group had slightly higher mean HbA1c (6.72 vs. 6.50%), lower mean total cholesterol (159.66 vs. 165.89 mg/dL) and lower mean LDL (89.18 vs. 93.56 mg/dL). Mean SBP, BMI, eGFR, and PSA were already well balanced between 2 groups with SMD ≤ 0.1 before PSM.

Following 1:1 PSM, two balanced cohorts (n = 32,918 per group) were established. All baseline covariates achieved adequate balance, as evidenced by an SMD of less than 0.1 for nearly all variables, ensuring the comparability of the two groups for subsequent analysis.

### 3.2. Primary Outcome

**Figure 2:**
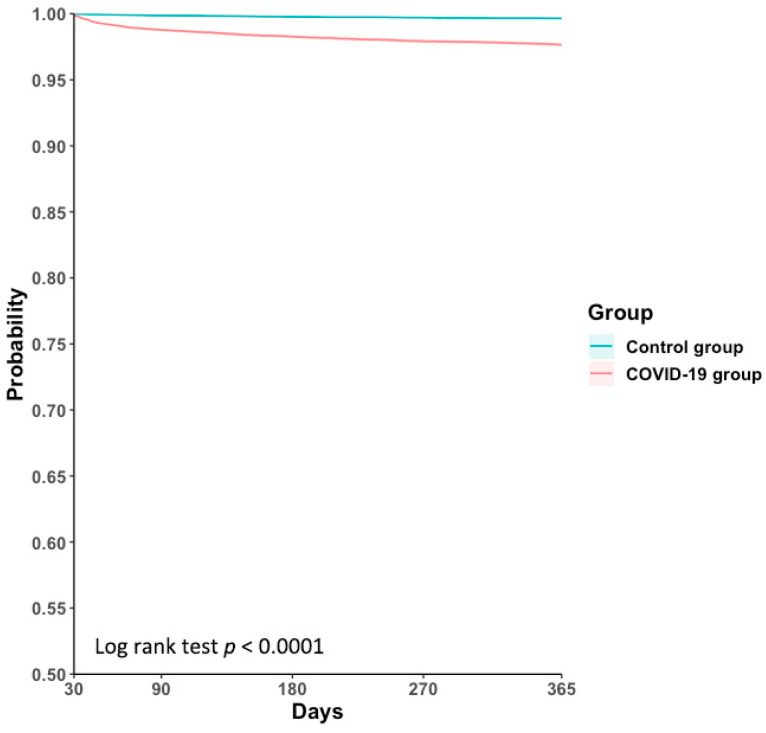
1-Year AUR-Free Survival in BPH Patients with LUTs: COVID-19 group vs. Control group after PSM. AUR: Acute Urinary Retention; BPH: Benign Prostatic Hyperplasia; COVID-19: Coronavirus Disease 2019; LUTs: Lower Urinary Tract Symptoms; PSM: Propensity Score Matching.

The 1-year cumulative incidence of AUR (Table 2) was significantly higher in the COVID-19 cohort (2.18%) compared to the matched control group (0.32%) with an adjusted hazard ratio (aHR) of 6.89 (95% CI, 5.62–8.45). The E-value of 5.82 also suggests a minimal strength of association between the unmeasured confounders and the observed outcome. Kaplan–Meier survival analysis (Figure 2) demonstrated a consistent divergence in AUR-free survival between the 2 groups over 1-year follow-up (Log rank test *p* < 0.0001).

**Figure 3:**
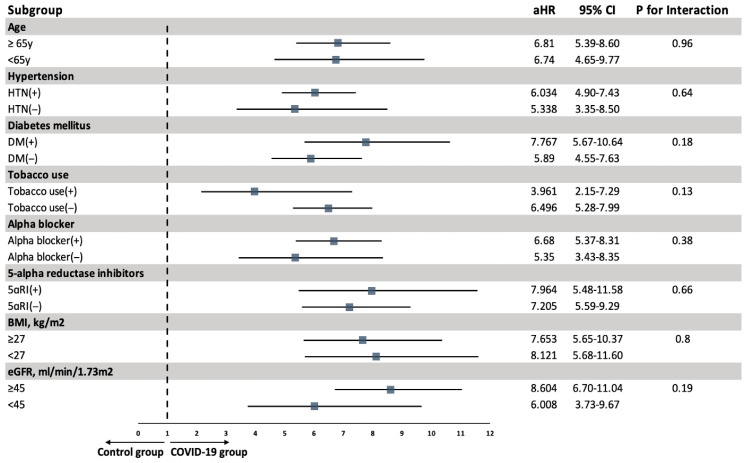
Subgroup analysis of AUR risk in BPH and LUT Patients with or without COVID-19. AUR: Acute Urinary Retention; BMI: Body Mass Index; BPH: Benign Prostatic Hyperplasia; COVID-19: Coronavirus Disease 2019; DM: Diabetes Mellitus; eGFR: Estimated Glomerular Filtration Rate; HTN: Hypertension; aHR: Adjusted Hazard Ratio; LUTs: Lower Urinary Tract Symptoms.

The elevated risk of AUR in the COVID-19 cohort remained remarkably stable across all strata (Figure 3). High aHRs persisted in subgroups of ≥65 vs. <65 years (6.81 vs. 6.74), presence vs. absence of HTN (6.034 vs. 5.338), presence vs. absence of DM (7.767 vs. 5.89), or smokers vs. non-smokers (3.961 vs. 6.496). Similar associations were observed in subgroups of alpha-blockers (user, aHR [6.68] vs. non-user, aHR [5.35]), 5-alpha reductase inhibitors (user, aHR [7.964] vs. non-user, aHR [7.205]), BMI (≥27, aHR [7.653] vs. <27, aHR [7.205] kg/m^2^), and eGFR (≥45, aHR [8.604] vs. <45, aHR [6.008] ml/min/1.73 m^2^).

### 3.3. Secondary Outcomes

Significant risk elevations in Foley insertion (aHR 4.10; 95% CI, 3.43–4.91), UTI (aHR 3.52; 95% CI, 3.17–3.92), and surgery (aHR 6.02; 95% CI, 4.39–8.26) in the COVID-19 group were also observed (Table 2). The high E-values for Foley insertion (4.06), UTI (3.82) and surgery (4.88) consistently indicate the extremely low substantial influence from the unmeasured confounders. Modest but statistically significant risk increases were also noted for gross hematuria (aHR 1.18) and bladder stones (aHR 1.25).

## 4. Discussion

In this large propensity score-matched cohort study, we observed that patients with pre-existing BPH and LUTs who developed COVID-19 had a higher incidence of AUR compared with matched controls. In addition to the increased occurrence of AUR, secondary outcomes including Foley catheter insertion, UTI, gross hematuria, bladder stone formation, and surgical intervention were also more frequent in the COVID-19 group. These findings suggest that SARS-CoV-2 infection may be associated with clinical decompensation in patients with underlying BPH rather than merely worsening subjective symptoms.

Our findings are consistent with the large population-based study by Liu et al., which included 17,986 propensity score-matched patients receiving alpha-blocker therapy for LUTs [17]. In that study, SARS-CoV-2 infection was associated with a higher incidence of urinary retention (4.55% vs. 0.86%) with a mean follow-up of 54–55 days. Increased rates of urinary tract infection, hematuria, and escalation to combination therapy were also reported. Importantly, the increased risk of urinary retention was observed regardless of COVID-19 severity, suggesting that even non-critical infection may adversely affect lower urinary tract function [17]. Compared with the prior study, our study provides a larger cohort with broader generalizability and longer follow-up duration. First, instead of focusing on patients receiving alpha-blocker monotherapy in a single region, our study encompasses a larger and more diverse population by utilizing TriNetX. Second, we extended the follow-up period to one year, utilizing Kaplan–Meier analysis to demonstrate the long-term risk of AUR post-infection. These findings suggest the need for clinicians to monitor BPH symptoms closely for at least one year after COVID-19 to optimize the timing of urological assessments or surgical interventions, thereby reducing the incidence of AUR.

Smaller clinical investigations provide additional functional insight into this relationship. Sanli et al. reported that BPH patients recovering from COVID-19 experienced increases in International Prostate Symptom Score (IPSS), decreases in maximum urinary flow rate (Qmax), and increases in postvoid residual urine (PVR), despite no significant change in prostate volume or PSA [13]. Similarly, Pavlović et al. observed post-COVID increases in IPSS, PSA, prostate volume, and residual urine in a cohort of 80 BPH patients, among whom 11.3% developed AUR. Residual urine was identified as an independent predictor of AUR (OR 1.039, *p* = 0.015), suggesting that impaired bladder emptying may represent an important intermediary mechanism [15]. Together, these studies indicate that SARS-CoV-2 infection may exacerbate LUTs and increase residual urine, potentially predisposing susceptible individuals to urinary retention.

Mechanisms involving renin–angiotensin system dysregulation, cytokine storm, interplay between the androgen receptor and transmembrane serine protease 2 (TMPRSS2), metabolic dysregulation, and endothelial dysfunction after SARS-CoV-2 infection may potentially explain this association [18]. SARS-CoV-2 gains cellular entry through binding of its spike protein to the angiotensin-converting enzyme 2 (ACE2) receptor, with viral entry facilitated by TMPRSS2, an androgen-regulated protein [19,20]. Both ACE2 and TMPRSS2 are expressed in prostatic epithelial cells, suggesting that prostate tissue may be potentially susceptible to viral interaction [19]. Besides, COVID-19 involves a complex interplay between systemic inflammation and a prothrombotic state [21]. A recent case report has revealed histological evidence of prostatic infarction, extensive necrosis, and microvascular thrombosis in a BPH patient with AUR following severe COVID-19 infection [22]. These observations suggest that the virus may trigger a complex interplay between systemic inflammation and thrombosis, potentially leading to vascular compromise within the prostate tissue.

In addition, the association between cytokine release and BPH progression has been previously reported [23]. Inflammatory reactions may contribute not only to prostatic hyperplasia but also to alterations in bladder sensation and detrusor function through cytokine release, leading to symptoms such as urinary frequency and urgency [23]. Other mechanisms including endothelial dysfunction [24,25] and metabolic dysregulation [26,27] might exacerbate pre-existing BOO or impair detrusor contractility. In our study, the consistency of elevated AUR risk across subgroup analyses might suggest that COVID-19 acts as a precipitating trigger superimposed on baseline vulnerability rather than being confined to a specific comorbidity profile.

From a clinical standpoint, these findings have practical implications. AUR represents a major complication of BPH that often requires emergency catheterization and may ultimately lead to surgical intervention. Given the global burden of COVID-19 and the high prevalence of BPH in aging men, even a modest increase in AUR risk may translate into a substantial healthcare impact. Clinicians should consider closer monitoring of urinary symptoms in patients with BPH following COVID-19 infection for at least one year, to optimize the timing of urological assessments or surgical interventions.

Several limitations warrant consideration. First, regarding residual confounding, despite utilizing PSM to balance the cohorts and E-values to assess the robustness of our outcomes, the retrospective nature of electronic health records cannot fully eliminate unmeasured bias. Factors such as lifestyle habits, socioeconomic status, and genetic markers remain uncaptured. Second, this study lacks several crucial urological parameters. Due to database constraints, urodynamic data (QMax, detrusor pressure), PVR volume, and prostate volume evolution were unavailable. Additionally, the absence of IPSS prevents the assessment of functional deterioration preceding the AUR event. Third, virological and clinical severity data were limited; the TriNetX database does not provide details on COVID-19 severity (asymptomatic vs. severe) or specific viral variants. Furthermore, defining COVID-19 exposure through both ICD coding and PCR data, while common in real-world studies, may introduce heterogeneity due to varying diagnostic practices across healthcare organizations. Fourth, regarding clinical interpretation, while certain secondary outcomes like hematuria and bladder stones achieved statistical significance, their clinical effect sizes were relatively modest (aHR 1.18–1.25). Our study primarily focuses on AUR, where the risk elevation was substantially more pronounced (aHR 6.89). Thus, these secondary results should be interpreted as supplementary to the main functional finding of acute urinary decompensation.

## 5. Conclusions

Our study suggests that COVID-19 infection is associated with an increased risk of AUR and adverse urological outcomes in patients with pre-existing BPH/LUTS. This association likely involves a multifactorial mechanism—driven by systemic inflammation, vascular injury, and AR-regulated pathways—that accelerates BPH progression. Consequently, clinicians should consider closer monitoring of urinary symptoms for at least one year following infection to optimize the timing of urological assessment or intervention. Further prospective studies are warranted to define causal pathways and optimal surveillance strategies.

## Figures and Tables

**Table 1:** Baseline Characteristics.

	Before PSM			After PSM		
	COVID-19 Group (N = 32,948)	Control Group (N = 434,123)	SMD	COVID-19 Group (N = 32,918)	Control Group (N = 32,918)	SMD
**Age**						
At index, mean (SD)	66.65 (10.39)	65.93 (9.73)	0.072	66.65 (10.38)	66.34 (9.97)	0.030
41–50 years, n (%)	1895 (5.8%)	24,271 (5.6%)	0.007	1894 (5.8%)	1954 (5.9%)	0.008
51–60 years, n (%)	6383 (19.4%)	85,096 (19.6%)	0.006	6380 (19.4%)	6431 (19.5%)	0.004
61–70 years, n (%)	11,184 (33.9%)	162,627 (37.5%)	0.073	11,177 (34.0%)	11,131 (33.8%)	0.003
≥71 years, n (%)	13,486 (40.9%)	162,129 (37.3%)	0.074	13,467 (40.9%)	13,402 (40.7%)	0.004
**Race**						
White, n (%)	23,267 (70.6%)	268,997 (62.0%)	0.184	23,244 (70.6%)	23,344 (70.9%)	0.007
Black, n (%)	4088 (12.4%)	38,149 (8.8%)	0.118	4082 (12.4%)	4058 (12.3%)	0.002
Asian, n (%)	1220 (3.7%)	20,267 (4.7%)	0.048	1220 (3.7%)	1215 (3.7%)	0.001
American Indian or Alaska Native, n (%)	149 (0.5%)	1353 (0.3%)	0.023	149 (0.5%)	135 (0.4%)	0.006
Native Hawaiian or Pacific Islander, n (%)	215 (0.7%)	2011 (0.5%)	0.025	215 (0.7%)	201 (0.6%)	0.005
Other Race, n (%)	916 (2.8%)	12,572 (2.9%)	0.007	915 (2.8%)	906 (2.8%)	0.002
Unknown Race, n (%)	3093 (9.4%)	90,774 (20.9%)	0.326	3093 (9.4%)	3059 (9.3%)	0.004
**Diagnosis**						
Hypertensive diseases, n (%)	12,581 (38.2%)	83,930 (19.3%)	0.426	12,551 (38.1%)	12,619 (38.3%)	0.004
Diabetes mellitus, n (%)	6735 (20.4%)	41,995 (9.7%)	0.305	6708 (20.4%)	6631 (20.1%)	0.006
Ischemic heart diseases, n (%)	4323 (13.1%)	25,002 (5.8%)	0.254	4297 (13.1%)	4187 (12.7%)	0.010
Overweight, n (%)	2854 (8.7%)	13,162 (3.0%)	0.242	2835 (8.6%)	2785 (8.5%)	0.005
Tobacco use, n (%)	329 (1.0%)	2006 (0.5%)	0.063	329 (1.0%)	292 (0.9%)	0.012
**Medications**						
Alpha blockers, n (%)	5575 (16.9%)	40,468 (9.3%)	0.227	5548 (16.9%)	5339 (16.2%)	0.017
5-alpha reductase inhibitors, n (%)	1421 (4.3%)	10,589 (2.4%)	0.104	1414 (4.3%)	1254 (3.8%)	0.025
**Laboratory**						
SBP, mmHg, mean (SD)	130.00 (18.02)	131.78 (17.84)	0.100	130.00 (18.02)	132.46 (17.89)	0.137
≥130 mmHg, n (%)	11,951 (36.3%)	77,670 (17.9%)	0.423	11,925 (36.2%)	12,001 (36.5%)	0.005
BMI, kg/m^2^, mean (SD)	30.02 (6.17)	29.64 (5.84)	0.063	30.02 (6.17)	30.12 (6.13)	0.017
≥30, kg/m^2^, n (%)	7701 (23.4%)	44,316 (10.2%)	0.358	7683 (23.3%)	7833 (23.8%)	0.011
27–30, kg/m^2^, n (%)	4359 (13.2%)	27,043 (6.2%)	0.238	4335 (13.2%)	4386 (13.3%)	0.005
24–27, kg/m^2^, n (%)	3846 (11.7%)	24,361 (5.6%)	0.217	3824 (11.6%)	3830 (11.6%)	0.001
<24, kg/m^2^, n (%)	2507 (7.6%)	15,429 (3.6%)	0.177	2494 (7.6%)	2488 (7.6%)	0.001
eGFR, mL/min/1.73 m^2^, mean (SD)	73.5 (27.3)	74.1 (24.2)	0.0259	73.5 (27.3)	72.7 (25.9)	0.032
≤45, mL/min/1.73 m^2^, n (%)	2650 (8.04%)	12,585 (2.90%)	0.228	2625 (7.97%)	2502 (7.60%)	0.014
HbA1c, %, mean (SD)	6.72 (1.53)	6.50 (1.36)	0.150	6.72 (1.53)	6.69 (1.44)	0.021
≥7, n (%)	2142 (6.5%)	12,537 (2.9%)	0.171	2127 (6.5%)	2059 (6.3%)	0.008
Total Cholesterol, mg/dL, mean (SD)	159.66 (42.74)	165.89 (40.75)	0.149	159.76 (42.72)	160.07 (41.18)	0.007
≥240, mg/dL, n (%)	255 (0.8%)	2451 (0.6%)	0.026	255 (0.8%)	235 (0.7%)	0.007
200–240, mg/dL, n (%)	764 (2.3%)	8137 (1.9%)	0.031	764 (2.3%)	724 (2.2%)	0.008
LDL, mg/dL, mean (SD)	89.18 (38.66)	93.56 (34.81)	0.119	89.27 (38.65)	88.84 (34.72)	0.012
≥160, mg/dL, n (%)	247 (0.8%)	2345 (0.5%)	0.026	247 (0.8%)	246 (0.7%)	0.000
100–160, mg/dL, n (%)	1914 (5.8%)	19,236 (4.4%)	0.063	1914 (5.8%)	1852 (5.6%)	0.008
PSA, ng/mL, mean (SD)	5.08 (37.70)	6.81 (564.69)	0.004	5.08 (37.70)	3.60 (10.84)	0.053
<4, ng/mL, n (%)	2712 (8.2%)	26,268 (6.1%)	0.085	2711 (8.2%)	2713 (8.2%)	0.000
4–20, ng/mL, n (%)	942 (2.9%)	11,650 (2.7%)	0.011	942 (2.9%)	921 (2.8%)	0.004
≥20, ng/mL, n (%)	68 (0.2%)	472 (0.1%)	0.025	67 (0.2%)	53 (0.2%)	0.010

BMI: Body Mass Index; COVID-19: Coronavirus Disease 2019; eGFR: Estimated Glomerular Filtration Rate; HbA1c: Hemoglobin A1c; LDL: Low-Density Lipoprotein; PSA: Prostate-Specific Antigen; PSM: Propensity Score Matching; SBP: Systolic Blood Pressure; SD: Standard Deviation; SMD: Standardized Mean Difference.

**Table 2:** Comparison of Outcomes in Patients with BPH and LUTs With and Without COVID-19.

	COVID-19 Group (N = 32,918)	Control Group (N = 32,918)	aHR (95% CI)	*p*-Value	E-Value (95% LCL)
**Primary Outcome**					
Acute urine retention	718 (2.18%)	106 (0.32%)	6.89 (5.62–8.45)	**<0.0001**	5.82
**Secondary Outcome**					
Foley insertion	598 (1.82%)	148 (0.45%)	4.10 (3.43–4.91)	**<0.0001**	4.06
UTI	1526 (4.64%)	444 (1.35%)	3.52 (3.17–3.92)	**<0.0001**	3.82
Gross hematuria	792 (2.41%)	680 (2.07%)	1.18 (1.06–1.30)	**0.0021**	1.25
Bladder stone	210 (0.64%)	169 (0.51%)	1.25 (1.02–1.53)	**0.0293**	1.13
Surgery	268 (0.81%)	45 (0.14%)	6.02 (4.39–8.26)	**<0.0001**	4.88

BPH: Benign Prostatic Hyperplasia; CI: Confidence Interval; COVID-19: Coronavirus Disease 2019; aHR: Adjusted Hazard Ratio; LCL: Lower Confidence Limit; LUTs: Lower Urinary Tract Symptoms; UTI: Urinary Tract Infection.

## Data Availability

The data used in this study were obtained from the TriNetX Research Network, a global federated network of de-identified electronic health records. Due to data privacy and institutional agreements, the datasets analyzed during the current study are not publicly available. However, they can be accessed through the TriNetX platform by authorized users.

## Supplementary Materials

The following supporting information can be downloaded at https://www.mdpi.com/article/10.3390/life16050729/s1. Table S1. Demographic, Diagnostic, Procedural, Medication, Visit, and Laboratory Codes Used in the Definition of the Cohorts; Table S2. Demographic, Diagnostic, and Laboratory Codes Used in the Definition of Covariates; Table S3. Diagnostic, Visit, and Procedural Codes Used in the Definition of Outcomes.

## Abbreviations

The following abbreviations are used in this manuscript:

ACE2	Angiotensin-Converting Enzyme 2
aHR	Adjusted Hazard Ratio
AUR	Acute Urinary Retention
BMI	Body Mass Index
BOO	Bladder Outlet Obstruction
BPH	Benign Prostatic Hyperplasia
CI	Confidence Interval
COVID-19	Coronavirus Disease 2019
CPT	Current Procedural Terminology
CVA	Cerebrovascular Accident
DM	Diabetes Mellitus
eGFR	Estimated Glomerular Filtration Rate
HbA1c	Hemoglobin A1c
HCOs	Healthcare Organizations
HTN	Hypertension
ICD-10	International Classification of Diseases, Tenth Revision
IPSS	International Prostate Symptom Score
IRB	Institutional Review Board
LDL	Low-Density Lipoprotein
LUTs	Lower Urinary Tract Symptoms
MS	Multiple Sclerosis
OR	Odds Ratio
PCR	Polymerase Chain Reaction
PCa	Prostate Cancer
PD	Parkinson’s Disease
PHEIC	Public Health Emergency of International Concern
PSA	Prostate-Specific Antigen
PSM	Propensity Score Matching
PVR	Postvoid Residual
Qmax	Maximum Urinary Flow Rate
SCI	Spinal Cord Injury
SD	Standard Deviation
SMD	Standardized Mean Difference
STROBE	Strengthening the Reporting of Observational Studies in Epidemiology
TMPRSS2	Transmembrane Serine Protease 2
UTI	Urinary Tract Infection
WHO	World Health Organization
5-ARI	5-Alpha Reductase Inhibitor
