# Supplementary material for: Elevated Risk of Acute Urine Retention in Patients with Symptomatic Benign Prostate Hyperplasia Following Coronavirus Disease 2019 Infection: A Retrospective Cohort Study from TriNetX
Source: Life (Basel). 2026 Apr 25;16(5):729. doi: 10.3390/life16050729 (PMC13208805; doi:10.3390/life16050729)
Supplement: Supplementary file 1 [file life-16-00729-s001.zip › life-4218302-supplementary.pdf]

Table S1. Demographic, Diagnostic, Procedural, Medication, Visit, and Laboratory Codes Used in the Definition of the Cohorts

Category	Code	Description
**COVID-19 group**		
**#1:** BPH with LUTs patients age ≥40 with COVID-19		
**#1.1:** Patients with BPH age ≥40<br>(Date constraint: The terms in this group occurred between Jan 30, 2020 and Jan 30, 2024)		
Diagnosis	UMLS:ICD10CM:N40.1	Benign prostatic hyperplasia with lower urinary tract symptoms (at least 40 years old at event)
**#1.2:** Patients with COVID-19(Must have any of the following)		
Laboratory	TNX:9088	SARS coronavirus 2 and related RNA [Presence] (labResult: Positive)
Diagnosis	UMLS:ICD10CM:U07.1	COVID-19
**#1.3:** No previous AUR, SCI, CVA, prostate cancer, urethral stricture, operation for BPH (Cannot have any of the following)<br>#1.3 should be fulfilled before #1.2		
Diagnosis	UMLS:ICD10CM:R33	Retention of urine
Diagnosis	UMLS:ICD10CM:N35	Urethral stricture
Diagnosis	UMLS:ICD10CM:G95	Spinal cord injury
Diagnosis	UMLS:ICD10CM:C61	Prostate cancer
Diagnosis	UMLS:ICD10CM:I60-I69	Cerebrovascular accident
Diagnosis	UMLS:ICD10CM:N39.0	Urinary tract infection
Diagnosis	UMLS:ICD10CM:G35	Multiple sclerosis
Diagnosis	UMLS:ICD10CM:G20	Parkinson's disease
Diagnosis	UMLS:ICD10CM:Z90.79	Prostatectomy
Procedure	UMLS:ICD10PCS:0VT08ZZ	Resection of Prostate, Via Natural or Artificial Opening Endoscopic
Procedure	UMLS:CPT:52648	Laser vaporization of prostate
Procedure	UMLS:CPT:52649	Laser enucleation of the prostate with morcellation
**#1.4:** No Foley insertion within previous 6 months of COVID-19 infection (Cannot have any of the following)<br>#1.4 should be fulfilled before #1.2		
Diagnosis	UMLS:ICD10CM:Z96.0	Presence of urogenital implants
Procedure	UMLS:CPT:51702	Insertion of temporary indwelling bladder catheter; simple (eg, Foley)
**Control group**		
**#1:** BPH with LUTs patients age ≥40 without COVID-19		
**#1.1:** Patients with BPH age ≥40<br>(Date constraint: The terms in this group occurred between Jan 30, 2020 and Jan 30, 2024)		
Diagnosis	UMLS:ICD10CM:N40.1	Benign prostatic hyperplasia with lower urinary tract symptoms (at least 40 years old at event)
**#1.2:** Patients without COVID-19 (Cannot have any of the following)		
Laboratory	TNX:9088	SARS coronavirus 2 and related RNA [Presence] (labResult: Positive)

**#1.3:** No previous AUR, SCI, CVA, prostate cancer, urethral stricture, operation for BPH (Cannot have any of the following)<br>#1.3 should be fulfilled before #1.1 and #1.2		
Diagnosis	UMLS:ICD10CM:R33	Retention of urine
Diagnosis	UMLS:ICD10CM:N35	Urethral stricture
Diagnosis	UMLS:ICD10CM:G95	Spinal cord injury
Diagnosis	UMLS:ICD10CM:C61	Prostate cancer
Diagnosis	UMLS:ICD10CM:I60-I69	Cerebrovascular accident
Diagnosis	UMLS:ICD10CM:N39.0	Urinary tract infection
Diagnosis	UMLS:ICD10CM:G35	Multiple sclerosis
Diagnosis	UMLS:ICD10CM:G20	Parkinson's disease
Diagnosis	UMLS:ICD10CM:Z90.79	Prostatectomy
Procedure	UMLS:ICD10PCS:0VT08ZZ	Resection of Prostate, Via Natural or Artificial Opening Endoscopic
Procedure	UMLS:CPT:52648	Laser vaporization of prostate
Procedure	UMLS:CPT:52649	Laser enucleation of the prostate with morcellation
**#1.4:** No Foley insertion within previous 6 months of COVID-19 infection (Cannot have any of the following)<br>#1.4 should be fulfilled before #1.1 and #1.2		
Diagnosis	UMLS:ICD10CM:Z96.0	Presence of urogenital implants
Procedure	UMLS:CPT:51702	Insertion of temporary indwelling bladder catheter; simple (eg, Foley)

Table S2. Demographic, Diagnostic, and Laboratory Codes Used in the Definition of Covariates

Category	Code	Description
Demographics	AI	Age at index
Demographics	2106-3	White
Demographics	1002-5	American Indian or Alaska Native
Demographics	2076-8	Native Hawaiian or Other Pacific Islander
Demographics	2054-5	Black or African American
Demographics	2028-9	Asian
Demographics	2131-1	Other Race
Demographics	UNK	Unknown Race
Demographics	2186-5	Not Hispanic or Latino
Demographics	UN	Unknown Ethnicity
Diagnosis	I10-I1A	Hypertensive diseases
Diagnosis	E08-E13	Diabetes mellitus
Diagnosis	E65-E68	Overweight
Diagnosis	I20-I25	Ischemic heart diseases
Diagnosis	Z72.0	Tobacco use
Medication	G04CA	Alpha blockers
Medication	G04CB	5-alpha reductase inhibitors
Laboratory	9085	Systolic blood pressure
Laboratory	9083	Body mass index
Laboratory	8001	Estimated glomerular filtration rate
Laboratory	9037	Hemoglobin A1c
Laboratory	9000	Total cholesterol
Laboratory	9002	Low density lipoprotein
Laboratory	9057	Prostate specific Antigen

Table S3. Diagnostic, Visit, and Procedural Codes Used in the Definition of Outcomes

Category	Code	Description
**#1:** Acute urine retention		
Diagnosis	UMLS:ICD10CM:R33	Retention of urine (principal Indicator: Primary Priority)
**#2:** Foley insertion (have any of the following)		
Procedure	UMLS:CPT:51702	Insertion of temporary indwelling bladder catheter; simple (eg, Foley)
Diagnosis	UMLS:ICD10CM:Z96.0	Presence of urogenital implants
**#3:** Urinary tract infection		
Diagnosis	UMLS:ICD10CM:N39.0	Urinary tract infection, site not specified
**#4:** Gross hematuria		
Diagnosis	UMLS:ICD10CM:R31.0	Gross hematuria
**#5:** Bladder stone		
Diagnosis	UMLS:ICD10CM:N21.0	Calculus in bladder
**#6:** Prostate surgery (have any of the following)		
Procedure	UMLS:ICD10PCS:0VT08ZZ	Resection of Prostate, Via Natural or Artificial Opening Endoscopic
Procedure	UMLS:CPT:52648	Laser vaporization of prostate, including control of postoperative bleeding, complete (vasectomy, meatotomy, cystourethroscopy, urethral calibration and/or dilation, internal urethrotomy and transurethral resection of prostate are included if performed)
Procedure	UMLS:CPT:52649	Laser enucleation of the prostate with morcellation, including control of postoperative bleeding, complete (vasectomy, meatotomy, cystourethroscopy, urethral calibration and/or dilation, internal urethrotomy and transurethral resection of prostate are included if performed)
